# The efficacy of femoral augmentation for hip fracture prevention using ceramic-based cements: A preliminary experimentally-driven finite element investigation

**DOI:** 10.3389/fbioe.2023.1079644

**Published:** 2023-01-26

**Authors:** Anita Fung, Ingmar Fleps, Peter A. Cripton, Pierre Guy, Stephen J. Ferguson, Benedikt Helgason

**Affiliations:** ^1^ Laboratory for Orthopaedic Technology, Institute for Biomechanics, Department of Health Sciences and Technology, ETH Zürich, Zürich, Switzerland; ^2^ Orthopaedic and Developmental Biomechanics Laboratory, Department of Mechanical Engineering, Boston University, Boston, MA, United States; ^3^ Orthopaedic and Injury Biomechanics Group, School of Biomedical Engineering and Departments of Mechanical Engineering and Orthopaedics, University of British Columbia, Vancouver, BC, Canada; ^4^ Centre for Hip Health and Mobility, University of British Columbia, Vancouver, BC, Canada; ^5^ Division of Orthopaedic Trauma, Department of Orthopaedics, University of British Columbia, Vancouver, BC, Canada

**Keywords:** mechanical properties, ceramic-based cement, computational models, impact, hip fracture

## Abstract

Femoral fractures due to sideways falls continue to be a major cause of concern for the elderly. Existing approaches for the prevention of these injuries have limited efficacy. Prophylactic femoral augmentation systems, particularly those involving the injection of ceramic-based bone cements, are gaining more attention as a potential alternative preventative approach. We evaluated the mechanical effectiveness of three variations of a bone cement injection pattern (basic ellipsoid, hollow ellipsoid, small ellipsoid) utilizing finite element simulations of sideways fall impacts. The basic augmentation pattern was tested with both high- and low-strength ceramic-based cements. The cement patterns were added to the finite element models (FEMs) of five cadaveric femurs, which were then subject to simulated sideways falls at seven impact velocities ranging from 1.0 m/s to 4.0 m/s. Peak impact forces and peak acetabular forces were examined, and failure was evaluated using a strain-based criterion. We found that the basic HA ellipsoid provided the highest increases in both the force at the acetabulum of the impacted femur (“acetabular force”, 55.0% ± 22.0%) and at the force plate (“impact force”, 37.4% ± 15.8%). Changing the cement to a weaker material, brushite, resulted in reduced strengthening of the femur (45.2% ± 19.4% acetabular and 30.4% ± 13.0% impact). Using a hollow version of the ellipsoid appeared to have no effect on the fracture outcome and only a minor effect on the other metrics (54.1% ± 22.3% acetabular force increase and 35.3% ± 16.0% impact force increase). However, when the outer two layers of the ellipsoid were removed (small ellipsoid), the force increases that were achieved were only 9.8% ± 5.5% acetabular force and 8.2% ± 4.1% impact force. These results demonstrate the importance of supporting the femoral neck cortex to prevent femoral fractures in a sideways fall, and provide plausible options for prophylactic femoral augmentation. As this is a preliminary study, the surgical technique, the possible effects of trabecular bone damage during the augmentation process, and the effect on the blood supply to the femoral head must be assessed further.

## 1 Introduction

Hip fractures in the elderly are associated with high socio-economic costs. By 2025, 810,000 fractures are expected to occur in the EU and cost 25.3 billion euros per year in direct medical costs (Hernlund et al., 2013). Hip fractures are associated with high morbidity and mortality rates (Fierens and Broos, 2006) and occur, in the vast majority of cases, due to sideways falls (Parkkari et al., 1999). At 6 months after a hip fracture, only 15% of survivors can walk across a room unaided (Marottoli et al., 1992). Furthermore, patients who have already sustained a hip fracture are more predisposed to having a second hip fracture (Sobolev et al., 2015), which often require more healthcare resources than the first hip fracture (Guy et al., 2017) and longer periods of immobilization (Ekström et al., 2009).

Existing treatments to prevent hip fractures include the use of pharmacological interventions to strengthen the femur and hip protectors which protect the femurs, but both have limited efficacy (Body et al., 2011; Järvinen et al., 2015). Cement-based interventions for the prophylactic augmentation of the proximal femur could overcome these limitations but require extensive evaluation to confirm that they would be safe, effective, durable and could be effectively introduced using surgical techniques that are safe for this patient population. The evaluation of the mechanical efficacy of polymer and ceramic cement-based femoral augmentations have been performed on cadavers (Heini et al., 2004; Beckmann et al., 2007; Sutter et al., 2010b; Sutter et al., 2010a; Beckmann et al., 2011; Fliri et al., 2013; Basafa et al., 2015; Stroncek et al., 2019). One of the main drawbacks of these important previous studies is in the method in which the strength of each femur is measured. In each study, each femur is loaded to fracture in order to measure its strength. These experiments measure the femoral strength without evaluating whether the subject-specific loads experienced during a sideways fall would actually surpass the femoral strength. It is also unclear how this method of measuring strength increases relative to unaugmented control femurs relates to a reduction in fracture risk because it does not take into account the subject-specific loading and fall probabilities (Parkkari et al., 1999; Choi et al., 2015). Additionally, it has been previously shown that the surrounding structures, such as the pelvis and soft tissue, absorb most of the impact energy in a sideways fall, and only 5%–10% of the energy is absorbed by the femur itself (Robinovitch et al., 1995; Laing and Robinovitch, 2010; Fleps et al., 2018a; 2019a). Furthermore, femoral augmentation might not only change femoral strength, but also toughness (i.e. energy absorbed prior to fracture). The increase in toughness alone could lead to reduced fracture risk. However, this reduction in risk can only be demonstrated by considering the impact force and energy due to a particular fall, which can result in impacts that do not fracture the femur. It follows that less than 5% of falls result in fractures (Nevitt et al., 1989; Hayes et al., 1996; Nachreiner et al., 2007). Without an estimate of the applied impact loading under consideration, a decrease in fracture risk due to an increase in toughness is challenging to quantify.

In order to address these shortcomings of the testing methods, a dynamic inertia-driven sideways fall simulator (Fleps et al., 2018b) has been developed, which includes the femurs, pelvis, and soft tissue surrogate. Advantages of the test setup include the use of subject-specific loading and the direct identification of fractures caused by sideways falls. Finite element models (FEMs) of the fall simulator have also been developed and validated, where they accurately predicted the fracture outcome in 10 of 11 specimens (Fleps et al., 2019b).

A previously-published *in silico* study using the FEMs of the novel sideways fall simulator (Fung et al., 2022) evaluated the mechanical efficacy of implants and found that although the implants increased the force sustained by the femur in a sideways fall prior to fracture, the femur still fractured at the highest impact velocities (3.1 m/s or higher). The results showed that the implants were not providing sufficient support to the weaker areas of the femur, such as at the femoral neck cortex, which is where the fractures typically initiate (de Bakker et al., 2009). The use of bone cements in these weaker areas could overcome these deficiencies because cements allow for the flexibility in both positioning and shaping. These attributes are useful for applications that require the direct support of the femoral neck cortex in three dimensions, such as for the prophylactic femoral augmentation under discussion here. In addition, the injection of relatively large volumes of ceramic-based bone cement is less concerning than for PMMA injections because of their good biocompatibility and absence of exothermic reactions that could damage tissue. Furthermore, the clinical feasibility of injections with large volumes into the proximal femur has previously been demonstrated (Howe et al., 2019; Stroncek et al., 2019).

Therefore, the aim of this study was to evaluate the strengthening effect of a ceramic-based bone cement pattern used for prophylactic femoral augmentation using FEMs of the novel fall simulator (Fleps et al., 2019b). The hypotheses for this study were: a) the augmented femurs would fracture for fewer of the simulated impacts and sustain a higher peak force in the inertia-driven simulations, b) changing the material of the bone cement would have a minor effect on the fracture outcome and peak forces, and c) making the cement pattern hollow would have a minor effect on the fracture outcome and peak forces.

## 2 Materials and methods

### 2.1 Baseline FEMs

The five specimens (1 female, 4 males) used in this analysis were previously described in another study (Fung et al., 2022), and are a subset of eleven specimens (6 females, 5 males) that were tested using an inverted-pendulum sideways fall simulator (Fleps et al., 2019a). The five specimens had exhibited femoral fractures in the fall simulator experiments and were thus identified as candidates that could benefit from prophylactic femoral augmentation. Therefore, the validated unaugmented FEMs for the five specimens were used as the controls in the present study.

**FIGURE 1:**
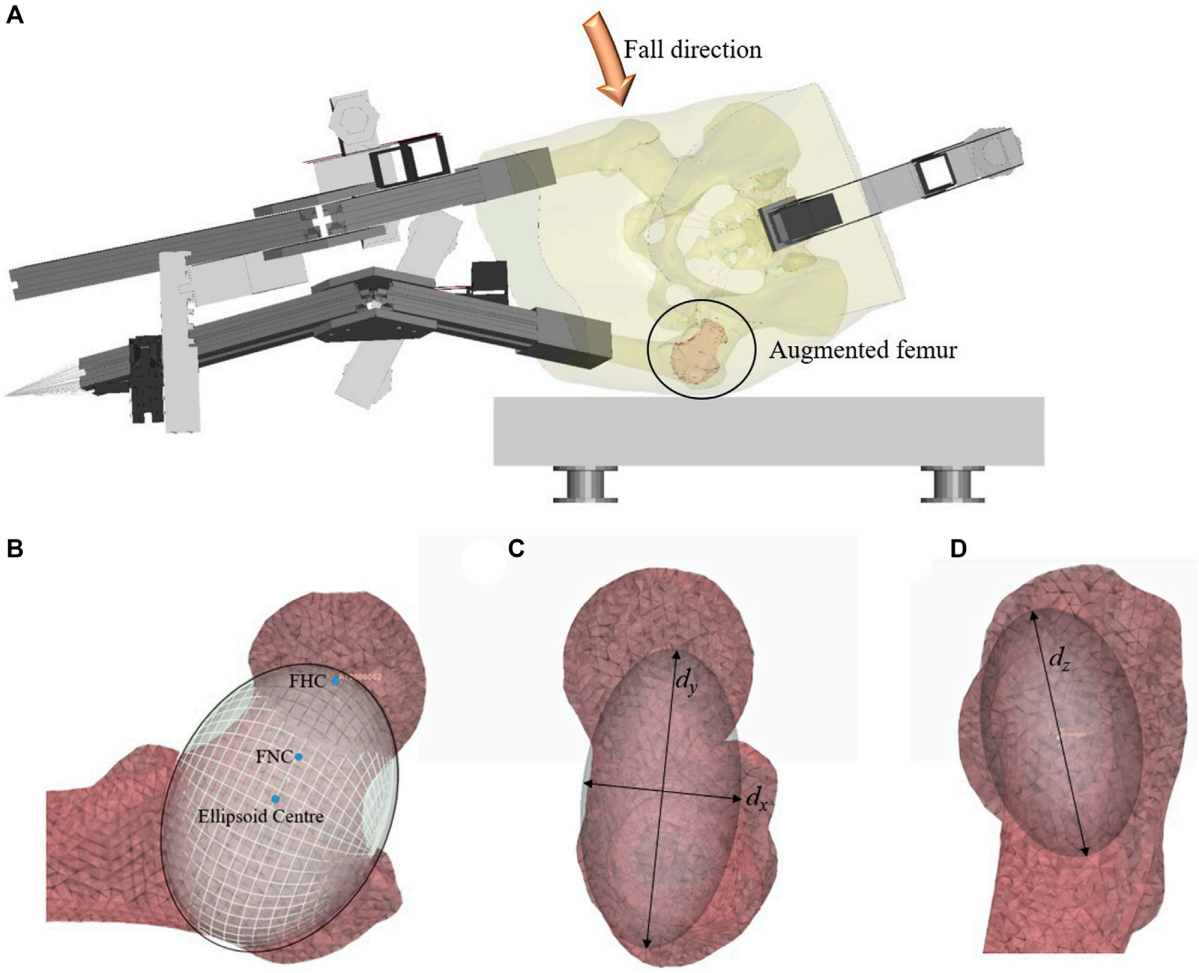
**(A)** The fall simulator FEM, **(B)** Positioning of the ellipsoid on the original mesh of the unaugmented femur, in a frontal cross-section, **(C)** an axial view, and **(D)** the view from the lateral side of the femur. The coordinate systems *d*_*x*_, *d*_*y*_, and *d*_*z*_ refer to the dimensions of the ellipsoid.

To construct the FEMs (Figure 1A), the geometries of each specimen’s pelvis and femurs were obtained using clinical-resolution CT scans (120 kVp, 200 mAs, voxel size: 0.78 mm × 0.78 mm × 0.3 mm). Descriptions of the lower limb geometry, soft tissue surrogate, cartilage, ligaments, and contact conditions have been previously published (Fleps et al., 2018a). Commercial FEM pre-processing software (Ansa 17.1.0, Beta CAE Systems, Switzerland) and solvers (LS-Dyna, Livermore, United States) were used to build the models.

### 2.2 Femoral augmentation

The cement augmentation pattern that was chosen for this study was determined by the positioning of an ellipsoid (Figures 1B–D) which provides a repeatable way to determine the placement of the bone cement. This pattern was informed by previous work which showed that the greater trochanter and femoral neck were susceptible to fracture during a sideways fall (Fleps et al., 2019a). In practice, this pattern would require the removal of the marrow and trabecular bone enclosed by the ellipsoid, followed by the filling of the cavity with bone cement.

**FIGURE 2:**
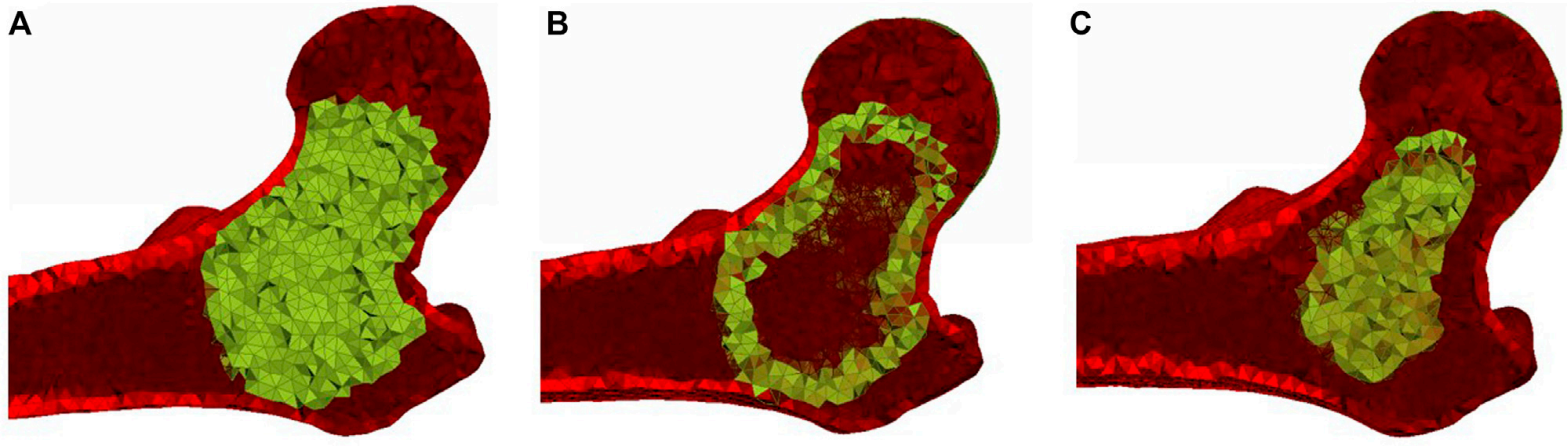
Planar cut views of **(A)** the basic ellipsoid cement pattern, **(B)** the hollow ellipsoid pattern, and **(C)** the small ellipsoid pattern.

For the FEMs, the cement augmentation pattern was created by placing an ellipsoid on the mesh of the unaugmented left femur. The long axis of the ellipsoid (*d*_*y*_) was aligned with the femoral neck axis and extended from the femoral head centre (FHC) to 5 mm beyond the cortex on the lateral side of the femur. The femoral neck axis was defined as the line extending from the femoral neck centre (FNC) to the FHC, with both points defined using the algorithm described in a previous publication (Enns-Bray et al., 2019). The dimension *d*_*x*_ of the ellipsoid was 1.5 times the width of the femoral neck at the FNC, and the dimension *d*_*z*_ of the ellipsoid was twice the width of the femoral neck at the FNC. The final ellipsoid shape and volume were adapted to the fit the intramedullary space at the femoral neck using the endosteal contour of the femur neck cortex of each specimen as its limit. This created the basic ellipsoid (Figure 2A). The dimensions and volumes of the constructed ellipsoids for each specimen are shown in Table 1.

**TABLE 1:** Femoral neck width and the dimensions and volume of the construction ellipsoid for each specimen. The dimensions *d*_*x*_, *d*_*y*_, and *d*_*z*_ are illustrated in Figure 1.

	H1389	H1397	H1399	H1401	H1406	Mean	SD
Femoral neck width at FNC (mm)	25.4	29.7	28.1	27.6	27.6	27.7	1.4
*d*_*x*_ (mm)	38.2	44.6	42.1	41.3	41.4	41.5	2.1
*d*_*y*_ (mm)	74.6	84.0	86.5	80.2	80.9	81.2	4.0
*d*_*z*_ (mm)	50.9	59.4	56.2	55.1	55.2	55.4	2.8
Volume (cm^3^)	75.8	116.6	107.3	95.6	96.9	98.4	13.7

To assign the cement properties, elements with nodes on the surface of the femur, as well as elements with an apparent density greater than 1.4 g/cm^3^ (Enns-Bray et al., 2018) were excluded for the identification of elements within the cement volume. The remaining elements inside the ellipsoid were assigned the mechanical properties of the bone cement (Figure 2A), either hydroxyapatite (HA) or brushite, as described in the Material Models section below. In order to determine the influence of bridging between different parts of the femoral cortex, additional injection patterns were developed that only included the two outermost layers of the identified elements for the cement assignment (hollow ellipsoid) in Figure 2B and a pattern that excluded the two outermost layers (small ellipsoid) in Figure 2C. The hollow ellipsoid uses a smaller volume of the augmentation material compared to the original pattern but still bridges between all parts of the femoral cortex, while the smaller filled ellipsoid does not connect to the femoral cortex directly. The volumes of the cement for the different ellipsoid patterns for each specimen are shown in Table 2.

**TABLE 2:** Cement volume of the cement ellipsoid patterns for each specimen.

	H1389	H1397	H1399	H1401	H1406	Mean	SD
Basic Ellipsoid (mL)	43.3	55.1	54.2	53.2	56.1	52.4	4.7
Hollow Ellipsoid (mL)	31.9	47.1	46.9	44.6	46.0	43.3	5.8
Small Ellipsoid (mL)	11.4	8.0	7.4	8.7	10.1	9.1	1.5

Table 2 reveals the potential confounding factor of the bone cement volume, as the cement volume of the hollow ellipsoid pattern exceeds the volume of the small ellipsoid pattern across all specimens. Therefore, a sub-study was done on all specimens to examine whether having a hollow ellipsoid with a lower cement volume than the small ellipsoid would affect the relative mechanical efficacy of the cement injection patterns. This sub-study required reducing the mesh size of the femur to 2 mm instead of 3 mm. The cement volumes in the sub-study are listed in Table 3.

**TABLE 3:** Cement volume of the cement ellipsoid patterns for the cement volume sub-study.

	H1389	H1397	H1399	H1401	H1406	Mean	SD
Basic Ellipsoid (mL)	44.4	68.8	57.8	57.2	71.3	59.9	10.8
Hollow Ellipsoid (mL)	18.2	33.4	24.9	21.8	27.4	25.1	5.7
Small Ellipsoid (mL)	26.2	35.5	32.8	35.4	43.9	34.8	6.4

### 2.3 Simulated sideways falls

The FEM and corresponding experiment has been described previously (Fleps et al., 2018a). In short, the model consists of an inertia-driven sideways fall simulator that rotates around the foot point and falls onto a force plate. The fall simulator comprises of a metal leg assembly with a cadaveric pelvis and proximal femur construction embedded in a soft tissue surrogate. The input velocities in the present study are the same as those used previously (Fung et al., 2022), and range between 1.0 m/s and 4.0 m/s. The input velocity for all simulations in the cement volume sub-study was 3.1 m/s, which was the same impact velocity for the original lab experiments using this inertia-driven fall model (Fleps et al., 2019a). The evaluated outputs of the FEMs include the impact force at the force plate, the force at the acetabulum of the impacted femur, and the fracture outcome of the specimen. Details of these outputs are described in Section 2.6.

### 2.4 Material models

The material mapping strategy for the bone tissue used in the present study has been described previously (Enns-Bray et al., 2018). Briefly, elements were assigned to 500 equally-spaced stiffness values between 0.01 GPa and 22 GPa. As in the previous models (Fung et al., 2022), the pelvis and contralateral femur was assigned strain rate dependent linear material properties, while the impacted femur had strain rate dependent non-linear material properties with tension-compression asymmetry. This was done to avoid having pelvic fractures as a confounding factor in the results. The femoral head shells described in the previous study (Fung et al., 2022) were also used in the present study.

**FIGURE 3:**
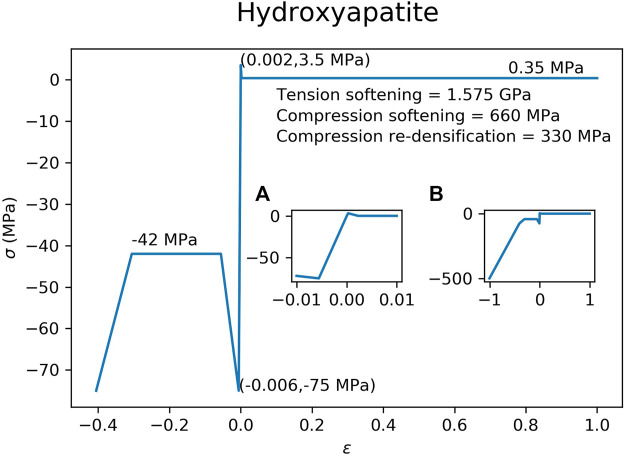
Material model implemented in the FEM for the hydroxyapatite cement. Inset figure **(A)** is a magnification of the elastic region of the curve, while inset figure **(B)** shows the curve at higher compressive strains.

**FIGURE 4:**
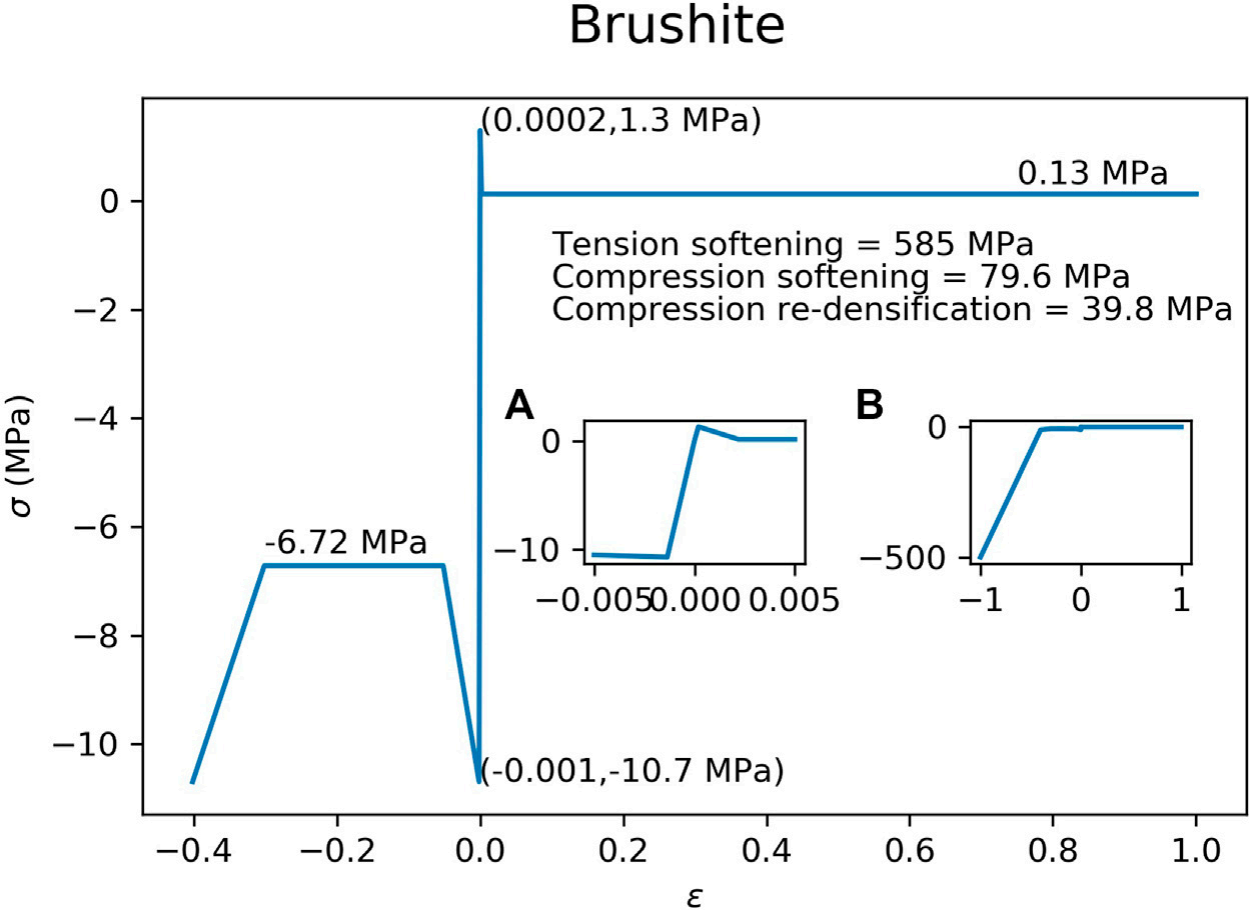
Material model implemented in the FEM for the brushite cement. Inset figure **(A)** is a magnification of the elastic region of the curve, while inset figure **(B)** shows the curve at higher compressive strains.

The material properties of the hydroxyapatite (Figure 3) and brushite (Figure 4) used in the FEM simulations were based on experimental test results (Charrière et al., 2001). A material model with tension-compression asymmetry (LS-DYNA, MAT 124) was implemented for both cement materials. After yielding, the compressive stress of the hydroxyapatite was decreased to 56.0% of the compressive strength (Figure 3), which corresponds to the reported porosity of 44.0% of the hydroxyapatite (Charrière et al., 2001). In a similar manner, the compressive stress of the brushite cement was decreased to 62.8% of its compressive strength (Figure 4), which corresponds to its porosity of 37.2% (Charrière et al., 2001). Densification was assumed for an additional 30% strain in compression for both cements. A similar implementation has already been used to successfully predict bone cement in cranial implant simulations (Lewin et al., 2020). FEMs using the HA properties were used for all three types of ellipsoids. The brushite material was used on only the basic ellipsoid pattern.

### 2.5 Cement-bone interface sensitivity analysis

The contact between the cement and the bone was modelled as fully tied under the assumption that a bond would be made (Tamimi et al., 2009; Draenert et al., 2013) through direct apposition and some interdigitation of remaining cancellous bone and cement. In the FEMs, this was modelled with the elements of the cement sharing nodes with the elements of the bone. To test the sensitivity of the cement-bone interface conditions, a worst-case frictionless interface test was run on the hydroxyapatite basic ellipsoid models at 3.1 m/s for all specimens. For the models with the frictionless cement-bone interface, a 0.01 mm gap was created between the cement elements and the bone elements to allow for movement of the cement.

### 2.6 Data processing

We calculated peak impact forces and peak acetabular forces (Fung et al., 2022). In addition, failure was evaluated using a strain-based criterion. Briefly, peak forces were estimated using virtual transducers at the acetabulum (“acetabular force”) and the surface of the force plate (“impact force”). Acetabular forces could be compared to the forces applied in femur-only studies (Beckmann et al., 2007; Sutter et al., 2010a; Stroncek et al., 2019), and the impact forces could be compared to experiments and simulations done on the sideways fall simulator (Fleps et al., 2019a; Fung et al., 2022).

The fragility ratio (FR) was also calculated by finding the ratio between the peak forces in the elastic simulation (FEMs_lin_) and corresponding non-linear simulation (FEMs_non-lin_). The maximum fragility ratio that a femur reaches before fracturing indicates how much the femur is relying on its ductility and toughness in the non-linear part of the loading curve. We used strain-based criteria to determine bone failure. The first and third principal engineering strains (LS-Dyna history variables #18 and #20) at 2 ms past the peak impact force were used to determine femoral fracture. The trabecular bone strain thresholds were 1.4% in tension and −2.0% in compression, while the cortical bone strain thresholds were 2.8% in tension and −5.9% in compression (Grassi et al., 2021). Bone failure was classified in three groups “no fracture”, a “trochanteric fracture”, and a “femoral neck fracture” (Fung et al., 2022).

## 3 Results

### 3.1 Peak forces and fragility ratios

The peak acetabular force increases across all specimens and impact velocities in which the femurs had fractured, were on average 55.0% (SD = 22.0%) for the basic HA ellipsoid, 45.2% (SD = 19.4%) for the basic brushite ellipsoid, 9.8% (SD = 5.5%) for the small HA ellipsoid, and 54.1% (SD = 22.3%) for the hollow HA ellipsoid. The peak impact force increases were 37.4% (SD = 15.8%) for the basic HA ellipsoid, 30.4% (SD = 13.0%) for the basic brushite ellipsoid, 8.2% (SD = 4.1%) for the small HA ellipsoid, and 35.3% (SD = 16.0%) for the hollow HA ellipsoid.

The results for the fragility ratios (Table 4) were also consistent with those for the peak forces as expected. No common fragility ratio threshold across specimens and augmentation conditions was apparent for the fragility ratios calculated with the acetabular force or the impact forces.

**TABLE 4:** Acetabular force fragility ratios. Green cells represent models exhibiting no femoral fractures, yellow cells represent fractures at the greater trochanter, and red cells represent femoral neck fractures as shown in (Fung et al., 2022).

a) Impact speed and corresponding acetabular force fragility ratio for specimen H1389.							
H1389	1.0 m/s	1.5 m/s	2.0 m/s	2.5 m/s	3.1 m/s	3.5 m/s	4.0 m/s
Unaugmented	1.0	1.1	1.6	2.0	2.4	2.7	3.0
HA ellipsoid	1.0	1.1	1.3	1.3	1.3	1.5	1.6
Brushite ellipsoid	1.0	1.1	1.3	1.4	1.5	1.6	1.8
HA small ellipsoid	1.0	1.2	1.5	1.9	2.3	2.5	2.8
HA hollow ellipsoid	1.0	1.1	1.3	1.3	1.3	1.5	1.6

b) Impact speed and corresponding acetabular force fragility ratio for specimen H1397.							
H1397	1.0 m/s	1.5 m/s	2.0 m/s	2.5 m/s	3.1 m/s	3.5 m/s	4.0 m/s
Unaugmented	1.0	1.0	1.1	1.2	1.3	1.4	1.6
HA ellipsoid	1.0	1.0	1.1	1.1	1.2	1.2	1.3
Brushite ellipsoid	1.0	1.0	1.1	1.1	1.2	1.2	1.3
HA small ellipsoid	1.0	1.0	1.1	1.2	1.3	1.4	1.5
HA hollow ellipsoid	1.0	1.0	1.1	1.1	1.2	1.2	1.2

c) Impact speed and corresponding acetabular force fragility ratio for specimen H1399.							
H1399	1.0 m/s	1.5 m/s	2.0 m/s	2.5 m/s	3.1 m/s	3.5 m/s	4.0 m/s
Unaugmented	1.1	1.2	1.3	1.5	1.7	1.9	2.1
HA ellipsoid	1.1	1.2	1.2	1.3	1.3	1.3	1.3
Brushite ellipsoid	1.1	1.2	1.2	1.3	1.3	1.3	1.3
HA small ellipsoid	1.1	1.2	1.2	1.4	1.5	1.7	1.9
HA hollow ellipsoid	1.1	1.2	1.2	1.3	1.3	1.3	1.3

d) Impact speed and corresponding acetabular force fragility ratio for specimen H1401.							
H1401	1.0 m/s	1.5 m/s	2.0 m/s	2.5 m/s	3.1 m/s	3.5 m/s	4.0 m/s
Unaugmented	1.0	1.0	1.2	1.4	1.7	1.8	2.0
HA ellipsoid	1.0	1.0	1.1	1.1	1.2	1.2	1.2
Brushite ellipsoid	1.0	1.0	1.1	1.1	1.2	1.2	1.2
HA small ellipsoid	1.0	1.0	1.2	1.3	1.5	1.6	1.7
HA hollow ellipsoid	1.0	1.0	1.1	1.1	1.2	1.2	1.2

e) Impact speed and corresponding acetabular force fragility ratio for specimen H1406.							
H1406	1.0 m/s	1.5 m/s	2.0 m/s	2.5 m/s	3.1 m/s	3.5 m/s	4.0 m/s
Unaugmented	1.0	1.1	1.2	1.4	1.7	1.9	2.1
HA ellipsoid	1.0	1.1	1.1	1.2	1.3	1.3	1.6
Brushite ellipsoid	1.0	1.1	1.1	1.2	1.2	1.3	1.5
HA small ellipsoid	1.0	1.1	1.1	1.2	1.5	1.7	1.9
HA hollow ellipsoid	1.0	1.1	1.1	1.2	1.3	1.4	1.6

### 3.2 Effect of bone cement material

**FIGURE 5:**
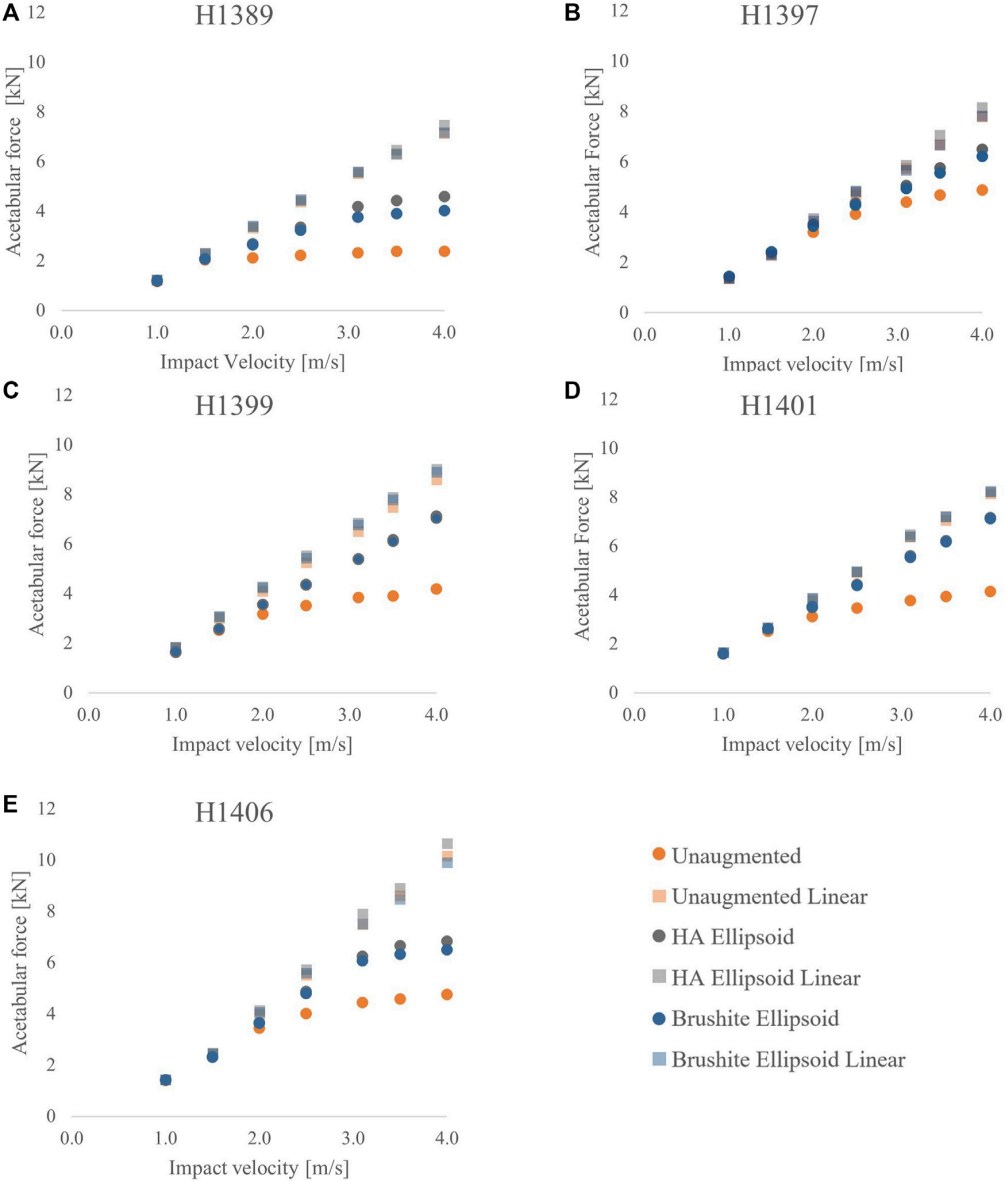
Acetabular force vs. impact velocity for the unaugmented, HA ellipsoid, and brushite ellipsoid pattern FEMs for each specimen: **(A)** H1389, **(B)** H1397, **(C)** H1399, **(D)** H1401, and **(E)** H1406.

The graphs on Figure 5 show that changing the cement material of the augmentation results in only small changes in the peak acetabular forces. In 4 of the 5 specimens, there was less than 5% difference between the peak forces of the HA ellipsoid augmentation and the brushite ellipsoid augmentation for any given specimen at any impact velocity. For higher impact velocities, the percent differences increased to a maximum of 13.2% for peak acetabular forces, and a maximum of 9.9% for peak impact forces.

### 3.3 Effect of bone cement pattern

In the cement volume sub-study, the basic ellipsoid augmentation FEM and the hollow ellipsoid FEM reached similar peak acetabular and impact forces (within 1.8% difference). Relative to the peak forces of the basic ellipsoid FEM, the peak acetabular force of the small ellipsoid FEM was on average 10.9% lower, and the peak impact force was on average 9.0% lower.

**FIGURE 6:**
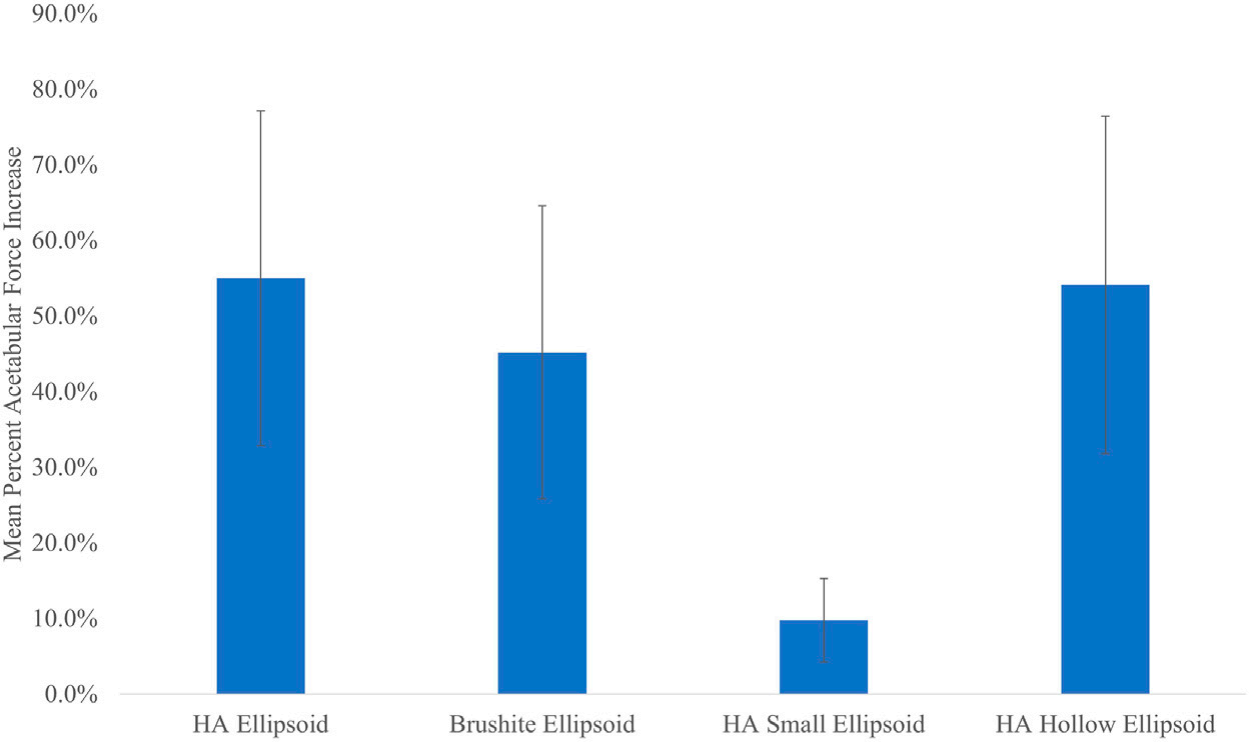
Mean percent acetabular force increase vs. cement pattern. Error bars indicate standard deviation.

Figure 6 shows that changing the size of the cement ellipsoid has a greater effect than making the cement pattern hollow. For all five specimens, both the peak acetabular forces and the peak impact forces were drastically reduced by reducing the size of the ellipsoid. However, the differences in the peak forces remain relatively unchanged if the ellipsoid is hollowed.

### 3.4 Fracture outcomes

Out of 35 non-linear simulations for each augmentation condition, femoral neck fractures appeared in 22 unaugmented femurs, 5 femurs augmented with the basic HA ellipsoid, 10 femurs augmented with the basic brushite ellipsoid, 17 femurs augmented with the small HA ellipsoid, and 5 femurs augmented with the HA hollow ellipsoid.

**FIGURE 7:**
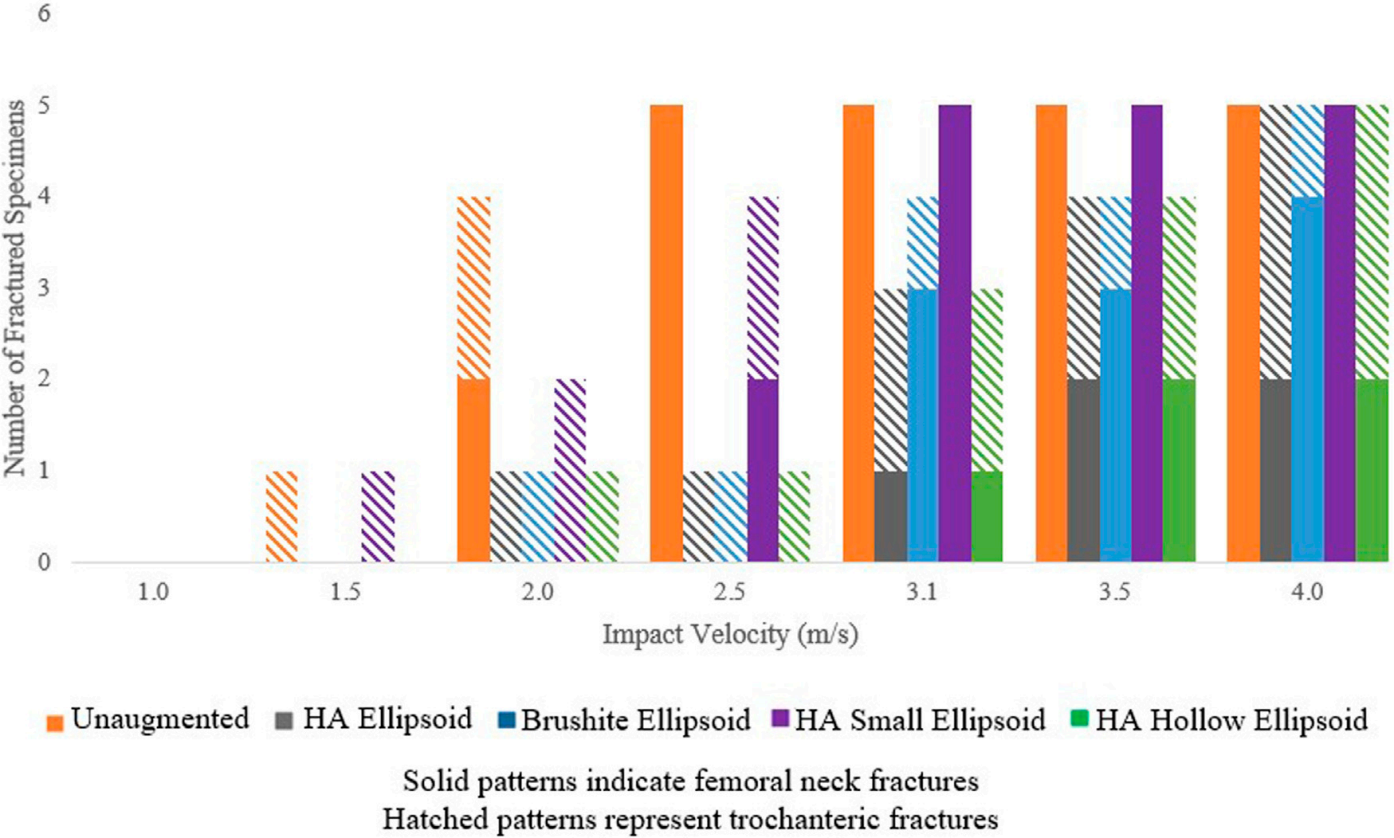
Number of fractured specimens vs. impact velocity as grouped by the type of fracture and augmentation pattern.

The breakdown of the fractured specimens by impact velocity and augmentation condition is shown in Figure 7. The predicted fracture outcome for the specimens augmented with the basic HA ellipsoid improved in all specimens at velocities up to 3.5 m/s. At 4.0 m/s, the femoral neck fractures for several specimens shifted to the trochanteric region. The first femoral neck fracture appeared at an impact velocity that was at least 0.5 m/s higher than in the unaugmented femur for all specimens. Changing the material of the cement to brushite had changed the fracture outcomes for 3 of the 5 specimens, reducing the impact velocity at which the first femoral neck fracture occurred. Reducing the size of the ellipsoid also resulted in slightly worse fracture outcomes than with the basic ellipsoid, but using a hollow ellipsoid did not change the fracture outcomes.

### 3.5 Effect of cement-bone interface conditions

**FIGURE 8:**
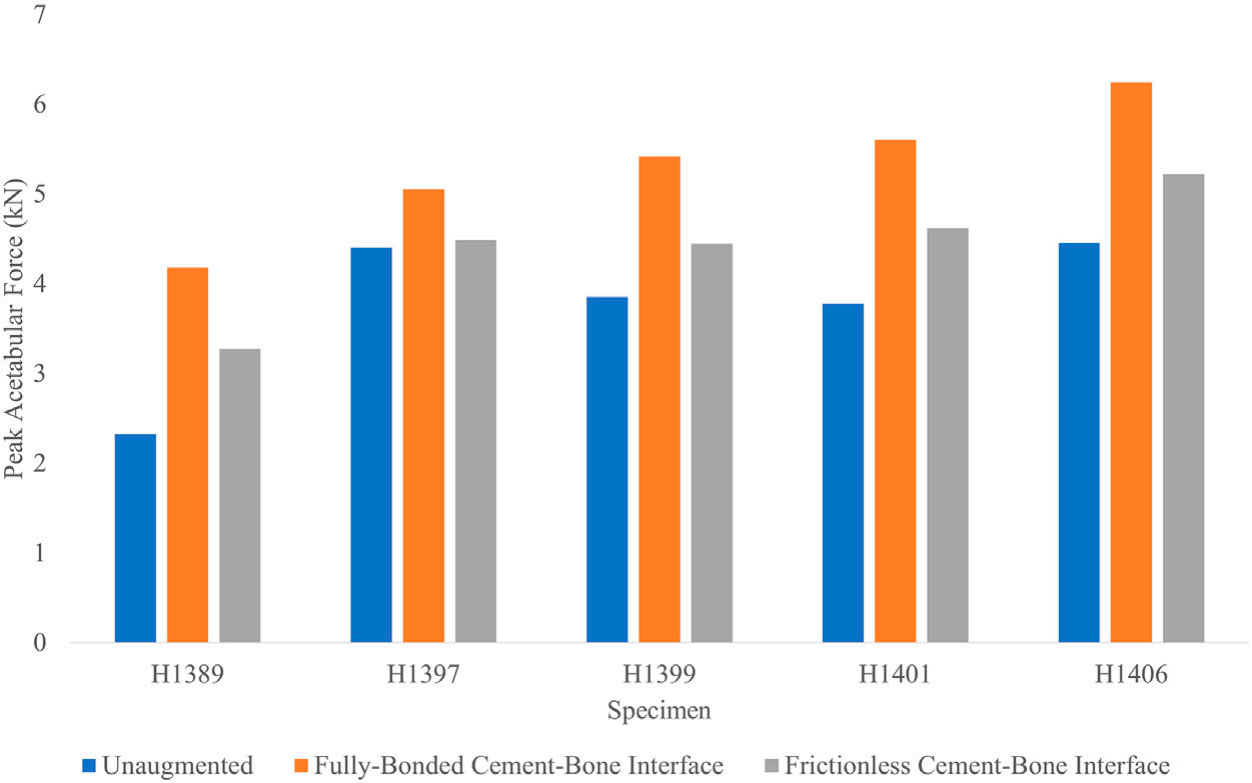
Peak acetabular forces for each specimen in the cement-bone interface sensitivity analysis.

The peak acetabular forces (Figure 8) of the basic HA ellipsoid FEMs at an impact velocity of 3.1 m/s and a frictionless cement-bone interface were on average 17.0% lower than the corresponding FEMs with the fully-bonded cement-bone interface. Of the five specimens, only H1399 had a worse fracture outcome with the frictionless cement-bone interface. On average, the acetabular force increase of the femurs augmented with the frictionless basic HA ellipsoid relative to the unaugmented FEMs was 19.5%. This is compared to 44.8% for the fully bonded basic HA ellipsoid augmentation at an impact velocity of 3.1 m/s.

Accordingly, the peak impact forces of the frictionless cement-bone interface FEMs were 13.2% lower than the corresponding FEMs with the fully-bonded interface. The average impact force increase of the frictionless interface FEMs were 13.8% relative to the unaugmented FEMs. This is compared to 31.4% for the FEM with the fully-bonded interface.

## 4 Discussion

The aim of this study was to evaluate the mechanical efficacy of a ceramic-based bone cement augmentation pattern using FEMs of a dynamic inertia-driven sideways fall simulator. The results showed that the acetabular force increases were the highest with the HA ellipsoid pattern and the hollow ellipsoid pattern, followed by the brushite ellipsoid pattern and the small ellipsoid pattern. Changing the cement to a weaker material reduced the peak forces and increased the number of fractures. Decreasing the size of the ellipsoid had a greater effect than making the cement pattern hollow. Having a weaker interface between the bone and the cement led to lower peak forces, but the augmented FEMs with a weaker cement-bone interface still had higher peak forces than the corresponding unaugmented femurs.

All specimens exhibited fractures at the highest velocity of 4.0 m/s, but the augmentation appears to shift some fractures from the femoral neck to the trochanteric region (Figure 7). In a clinical setting, a trochanteric fracture would be a better outcome than a fracture at the neck or at the trochanter, as they are most often minimally displaced or non-displaced and do not require surgery.

In this study, the maximum impact speed at which most of the augmented femurs did not fracture was 3.5 m/s, which was with the basic HA ellipsoid, the basic brushite ellipsoid, and the hollow HA ellipsoid. Comparing these values to the impact speeds reported in real-life falls (Choi et al., 2015), which had a mean of 2.14 m/s (SD = 0.63), this suggests that the augmentation could prevent femoral fractures at impact speeds with a maximum of two standard deviations above the mean impact speed.

In the present study, the increase in force that the femurs could support for the basic HA and brushite ellipsoid patterns were similar to the strength increases in some femur-only tests using femurs augmented in similar PMMA patterns (Beckmann et al., 2007; Sutter et al., 2010a). However, there was one study (Heini et al., 2004) which reported a strength increase for a PMMA-augmented femur which was substantially higher than the acetabular force increases found in the present study. Compared to experimental studies that examined resorbable ceramic-based cement, the peak acetabular force increases were higher than the strength increases reported previously (Howe et al., 2019; Stroncek et al., 2019). The peak acetabular force increases for the small HA ellipsoid were similar to the values reported in a study using FEMs with cement patterns with similar cement volumes (Kok et al., 2019). These differing results suggest that while the fracture strengths from the previous studies may be correlated to the acetabular peak forces in the present study, the values may not be directly comparable. It is also difficult to compare the present results to those from previous studies because the results might also depend on the fact that the present and previous studies capture the subject dependency of different femurs.

The results of the cement volume sub-study showed that the cement location is more important for femoral augmentation than the injected cement volume. In the sub-study, the cement volume for the hollow ellipsoid was approximately 60% of the volume of the basic ellipsoid, yet the peak forces between the two patterns were within only 1.8%. However, the small ellipsoid pattern, which had a higher cement volume than the hollow ellipsoid pattern, had lower peak forces of approximately 10%. This could be because the basic cement ellipsoid and the hollow ellipsoid support the cortical bone of the femoral neck by being in direct contact with the endosteal side of the cortex. This is in contrast with the implants tested previously (Fung et al., 2022), which had one rod going into the femoral neck. This finding is further supported by the results of the smaller ellipsoid, which did not have any contact with the femoral neck cortex. Removing the contact with the cortical bone had a negative effect on the fracture outcome, and also reduced the percent force increases and increased the fragility ratio. When the basic ellipsoid was made hollow, keeping only the cement elements that were in contact with the femoral neck cortex, the fracture outcomes were not affected, and only minor changes in the fragility ratio and force increases were observed. These results suggest that in order to prevent femoral fractures from sideways falls, it is important to support and thus stiffen or strengthen the cortex of the femoral neck.

Another factor that influences the mechanical efficacy of the bone cement injections is the material properties of the cement. When the properties of the HA basic ellipsoid were changed to a relatively weaker material, brushite, the fracture outcomes changed for 3 of the 5 specimens, with the femurs augmented with brushite failing at lower impact velocities. However, using the brushite ellipsoid still had improved fracture outcomes over the unaugmented femur and the femurs augmented by the implants tested in the previous study (Fung et al., 2022). These results demonstrate that for the best augmentation outcomes, it is also important to select a cement with strong tensile and compressive properties.

The results of the sensitivity test of the cement-bone interface conditions show that having no bonding between the cement and bone would decrease the peak acetabular forces by almost 17%. However, the increase of the peak acetabular forces of almost 20% relative to the unaugmented femurs shows that an augmentation with a weak cement-bone interface could still increase the load-bearing capacity of the femur.

In order to implement the cement ellipsoid in a femur, it is necessary to remove the cancellous bone occupying the space of the ellipsoid before injecting the cement. Table 2 shows that for the basic ellipsoid pattern, an average of over 50 mL of cement is required, which corresponds to an average of more than 50 mL of bone and marrow to be removed. One advantage of using ceramic-based cements instead of polymer cements such as PMMA is that these large volumes of cement could be injected without having temperature changes due to exothermic curing as a concern. Although the hollow ellipsoid provides an alternative for reducing the volume of cement without compromising the fracture outcome or mechanical strengthening effects, the pattern may be difficult to implement in practice.

Another practical consideration is the suitability of the cements for femoral augmentation. Although the two cements tested in this study have appropriate mechanical properties, both have shortfalls in either the injectability or the resorbability of the cement. Hydroxyapatite has a relatively slow resorption rate but is not injectable, while brushite is injectable but resorbs in 24 weeks (Charrière et al., 2001; Oberle et al., 2005). Although non-resorbable cements such as calcium aluminium phosphate are currently used in biomedical applications (Medri et al., 2011), the material properties are not well-described in the literature. Thus, further investigation into the development of fully non-resorbable ceramic-based cements with the appropriate mechanical properties would be recommended.

The present study has the limitation of having only one female specimen due to the fracture outcomes of the original validation study with the full set of specimens (Fleps et al., 2019a). Future studies should include more female specimens due to the higher likelihood of femoral fractures in females (Schuit et al., 2004; Haleem et al., 2008). Additionally, there is the limitation of a low overall number of specimens. It is possible that there are femurs that are not represented in this study in which it is very difficult to prevent fractures. Nevertheless, the results show that even in those cases, the load bearing capacity of the femurs will be improved with the augmentation, and the fragility ratio will be decreased, which shows that the femur is strengthened by the augmentation. Limitations of the FEMs are described in another study (Fleps et al., 2019b). They include the lack of an upper body, and homogeneity of the soft tissue, and the rigid attachment of masses in the leg assembly. An assumption that was made in the present study was the full osseointegration between the cement and the bone. This assumption was made because both HA and brushite cement have been shown to have good osseointegration, which provides instantaneous strength to the bone (Schliephake et al., 1991; Tamimi et al., 2009). Another limitation is the lack of experimental validation data for specimens augmented with cement tested using the novel fall simulator that the FEM is based on. Therefore, it is important that this study is followed up with an experimental validation study.

## 5 Conclusion

In summary, we found that a hydroxyapatite bone cement ellipsoid in contact with the endosteal side of the femoral neck cortex was able to prevent femoral fractures in finite element models of five cadaveric specimens for up to two standard deviations above the mean impact speed (Choi et al., 2015). The augmentation increased the peak forces supported by the femurs and decreased the fragility ratios. Changing the cement to a weaker material, brushite, reduced the impact speed at which the first femoral fracture appeared, but was still effective at preventing some femoral fractures. Making the ellipsoid hollow had no effect on the fracture outcomes, but reducing the size of the ellipsoid to remove contact with the cortex decreased the effectiveness of the augmentation. The results suggest that ceramic-based cements could be a viable option for prophylactic femoral augmentation for hip fracture prevention. Experimental validation of the augmented femur FEMs and further development of the cements and surgical techniques are recommended to optimize the treatment.

## Data Availability

The raw data supporting the conclusion of this article will be made available by the authors, without undue reservation.
